# HSD17B6 downregulation predicts poor prognosis and drives tumor progression via activating Akt signaling pathway in lung adenocarcinoma

**DOI:** 10.1038/s41420-021-00737-0

**Published:** 2021-11-08

**Authors:** Tian Tian, Fu Hong, Zhiwen Wang, Jiaru Hu, Ni Chen, Lei Lv, Qiyi Yi

**Affiliations:** 1 Department of Respiratory Oncology, Anhui Cancer Hospital, The First Affiliated Hospital of USTC, Division of Life Sciences and Medicine, University of Science and Technology of China, Hefei, Anhui 230031 People’s Republic of China; 2 Department of Radiation Oncology, Anhui Cancer Hospital, The First Affiliated Hospital of USTC, Division of Life Sciences and Medicine, University of Science and Technology of China, Hefei, Anhui 230031 People’s Republic of China; 3 The First Affiliated Hospital of USTC, Division of Life Sciences and Medicine, University of Science and Technology of China, Hefei, 230001 China; 4 School of Basic Medical Sciences, Anhui Medical University, 81 Meishan Road, Hefei, Anhui 230032 People’s Republic of China; 5 Department of Cancer Epigenetics Program, Anhui Cancer Hospital, The First Affiliated Hospital of USTC, Division of Life Sciences and Medicine, University of Science and Technology of China, Hefei, Anhui 230031 People’s Republic of China

**Keywords:** Non-small-cell lung cancer, Prognostic markers, Tumour-suppressor proteins

## Abstract

Lung adenocarcinoma is one of the most frequent tumor subtypes, involving changes in a variety of oncogenes and tumor suppressor genes. Hydroxysteroid 17-Beta Dehydrogenase 6 (HSD17B6) could synthetize dihydrotestosterone, abnormal levels of which are associated with progression of multiple tumors. Previously, we showed that HSD17B6 inhibits malignant progression of hepatocellular carcinoma. However, the mechanisms underlying inhibiting tumor development by HSD17B6 are not clear. Moreover, its role in lung adenocarcinoma (LUAD) is yet unknown. Here, we investigated its expression profile and biological functions in LUAD. Analysis of data from the LUAD datasets of TCGA, CPTAC, Oncomine, and GEO revealed that HSD17B6 mRNA and protein expression was frequently lower in LUAD than in non-neoplastic lung tissues, and its low expression correlated significantly with advanced tumor stage, large tumor size, poor tumor differentiation, high tumor grade, smoking, and poor prognosis in LUAD. In addition, its expression was negatively regulated by miR-31-5p in LUAD. HSD17B6 suppressed LUAD cell proliferation, migration, invasion, epithelial–mesenchymal transition (EMT), and radioresistance. Furthermore, HSD17B6 overexpression in LUAD cell lines enhanced PTEN expression and inhibited AKT phosphorylation, inactivating downstream oncogenes like GSK3β, β-catenin, and Cyclin-D independent of dihydrotestosterone, revealing an underlying antitumor mechanism of HSD17B6 in LUAD. Our findings indicate that HSD17B6 may function as a tumor suppressor in LUAD and could be a promising prognostic indicator for LUAD patients, especially for those receiving radiotherapy.

## Background

More than 2.2 million patients were diagnosed with lung cancer in 2020, making it the second most common tumor worldwide. It caused about 1.8 million deaths in 2020 globally and ranked as the first leading cause of cancer-associated death [1]. Approximately 50% of lung cancers are LUAD, which is the most common subtype [2]. Although new therapeutic strategies, such as targeted therapies, have achieved remarkable improvements in recent years, LUAD is still one of the most aggressive and fatal tumor types with overall survival <5 years [3], partly due to its usually invasive and metastatic activity [4]. For example, it can spread to bones and brain, escaping from surgery and treatment [5, 6]. The aggressiveness is often driven by molecular genetic/epigenetic abnormalities in LUAD. Efforts to identify these abnormalities will help to aid in the diagnosis and improve treatment of LUAD.

HSD17B6 encodes a protein named Hydroxysteroid 17-Beta Dehydrogenase 6. It could convert 3a-androstanediol to dihydrotestosterone (DHT), which is the most potent form of androgen [7, 8]. Dysregulation of DHT has been reported to affect the development of multiple tumors, such as prostate cancer and breast cancer [9]. Previous studies suggested that HSD17B6 might inhibit tumor progression. For example, HSD17B6 was significantly downregulated in prostate cancers with bone metastases than in non-metastatic primary tumors [10]. Its expression levels were downregulated in NSCLC (non-small cell lung cancer) than in non-tumorous lung tissues [11]. And we have reported that HSD17B6 expression was significantly lower in hepatocellular carcinoma [12]. However, the detailed mechanism by which HSD17B6 influences cancer progression in LUAD was not investigated. It is also unclear how HSD17B6 regulates invasion and metastasis in cancer cells.

In the present study, we characterized HSD17B6 expression and its regulation in LUAD tissues. We studied the effect of HSD17B6 on LUAD progression and explored the potential molecular mechanism. Our results may shed light on the role of HSD17B6 in the initiation and progression of LUAD and help develop potential strategies to inhibit LUAD metastasis.

## Results

### HSD17B6 expression is downregulated in LUAD tissues

**Fig. 1:**
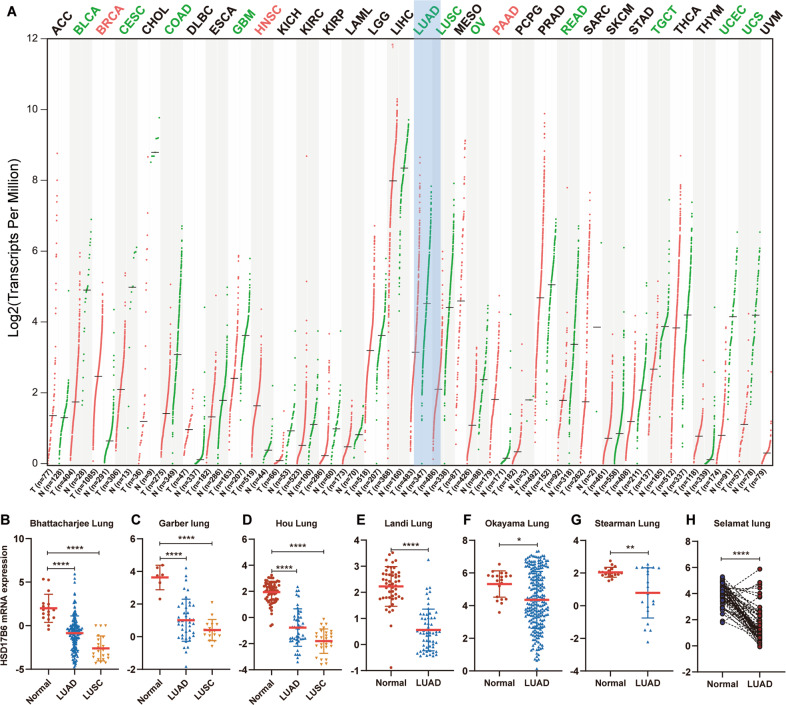
Differential mRNA expression of HSD17B6 between tumors and corresponding non-cancerous tissues. **A** Comparison of HSD17B6 mRNA levels between tumors and normal samples in 32 types of cancers in TCGA using the GEPIA web tool (red lines indicate tumor samples; green lines indicate non-cancerous samples). **B**–**H** Comparison of HSD17B6 mRNA expression among LUAD, LUSC, and adjacent non-cancerous tissues in 7 lung cancer datasets from Oncomine. LUAD lung adenocarcinoma, LUSC lung squamous cell carcinoma. ****p < 0.0001, **p < 0.01, *p < 0.05.

In order to investigate the potential role of HSD17B6 in tumors, We first analyzed the differential expression of HSD17B6 between tumor tissues and corresponding non-tumor tissues in the 32 cancers included in TCGA (http://gepia.cancer-pku.cn) [13]. The analysis revealed that HSD17B6 expression was substantially lower in 11 kinds of tumors compared to the non-tumor tissues, including LUAD (Lung Adenocarcinoma), LUSC (Lung Squamous Cell Carcinoma), and so on (Fig. 1A). Analysis of seven oncomine lung cancer datasets also showed that HSD17B6 mRNA levels were significantly reduced in LUAD compared with adjacent/healthy lung tissues (Fig. 1B–H). In addition, protein levels of HSD17B6 were significantly lower in LUAD than adjacent para-cancerous tissues by analyzing CPTAC (Clinical Proteomic Tumor Analysis Consortium) LUAD dataset (*p* < 0.0001, Fig. S1A), which was in accordance with the analysis of mRNA expression. Thus, these findings indicate that LUAD exhibits low levels of HSD17B6.

### Low HSD17B6 expression correlates with poor clinicopathological features in lung adenocarcinoma

**Fig. 2:**
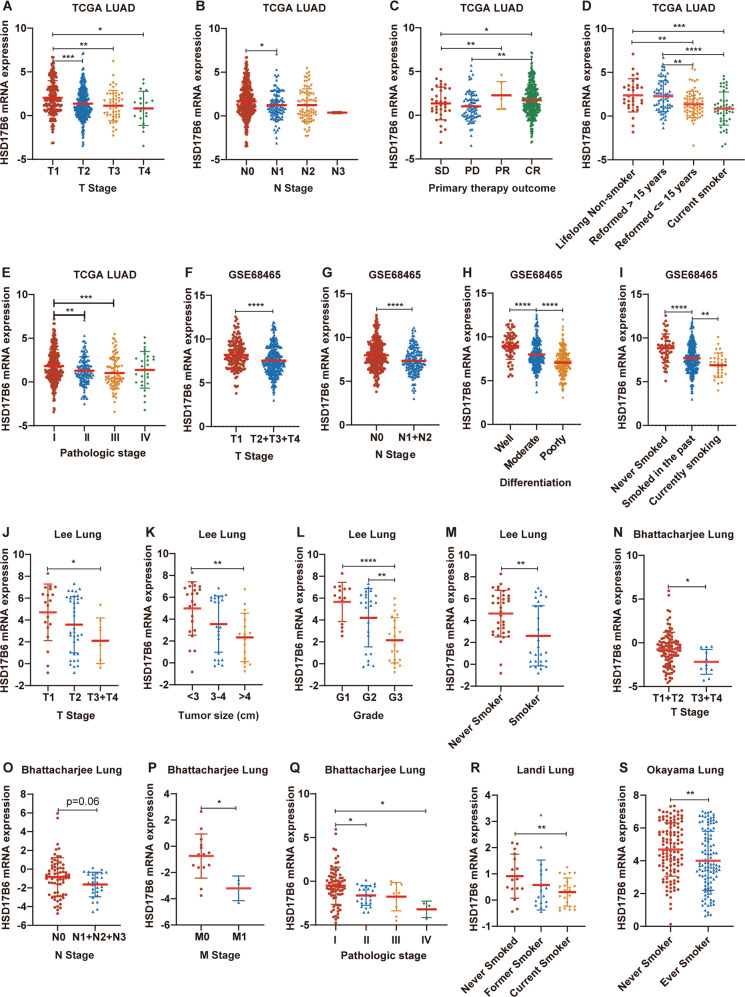
HSD17B6 mRNA levels decrease with LUAD progression. **A**–**E** HSD17B6 mRNA levels in different T stages (**A**), N stages (**B**), primary therapy outcome (**C**), smoking status (**D**), and pathologic stages (**E**) of LUAD samples in TCGA LUAD dataset. **F**–**I** HSD17B6 mRNA levels in different T stages (**F**), N stages (**G**), tumor differentiation (**H**), and smoking status (**I**) of LUAD samples in GSE68465 dataset. **J**–**M** HSD17B6 mRNA levels in different T stages (**J**), tumor size (**K**), grade (**L**), and smoking status (**M**) of LUAD samples in “Lee Lung” dataset. **N**–**Q** HSD17B6 mRNA levels in different T stages (**N**), N stages (**O**), M stages (**P**), and pathological stages (**Q**) of LUAD samples in “Bhattacharjee Lung” dataset. **R**, **S** HSD17B6 mRNA levels in LUAD samples from patients with different smoking status in “Landi lung” (**R**) and “Okayama lung” (**S**) datasets. ****p < 0.0001, ***p < 0.001, **p < 0.01, *p < 0.05.

Next, we investigate the association of HSD17B6 expression and the clinical characteristics of LUAD in multiple LUAD datasets, including TCGA LUAD and 6 datasets from oncomine and GEO databases. Decreased HSD17B6 mRNA expression was associated with advanced T stage (Table S1, Fig. 2A–F, J, N, data from TCGA LUAD, GSE68465, “Lee lung” and “Bhattacharjee Lung” datasets, respectively) in LUAD. Consistent with this result, HSD17B6 expression was also lower in large tumors than in small tumors (Fig. 2K). Its low expression was also associated with advanced N stage (Table S1, Fig. 2B, G, O, data from TCGA LUAD, GSE68465 and “Bhattacharjee Lung” datasets, respectively), M stage (Fig. 2P, data from “Bhattacharjee Lung” dataset), advanced pathologic stage, poor differentiation and high tumor grade in LUAD (Table S1, Fig. 2E, H, L, data from TCGA LUAD, GSE68465 and “Lee Lung” datasets, respectively). Moreover, its expression levels were remarkably lower in LUAD samples from smokers than those from non-smokers (Table S1, Fig. 2D, I, M, R, S, data from TCGA LUAD, GSE68465, “Lee lung”, “Landi lung”, and “Okayama lung” datasets, respectively). HSD17B6 expression in LUAD nonresponding to primary therapy (SD and PD) was lower than responding LUAD (CR and PR) (Table S1, Fig. 2C). In addition, decreased protein expression levels of HSD17B6 were also associated with advanced T/M/N stage and high grade in CPTAC LUAD dataset (Fig. S1B–E). And protein levels of HSD17B6 were considerably lower in large tumors than in small tumors (Fig. S1F).

These results collectively indicate that reduced HSD17B6 expression is associated with poor clinicopathological features in LUAD.

### HSD17B6 is a prognostic marker for lung adenocarcinoma

**Fig. 3:**
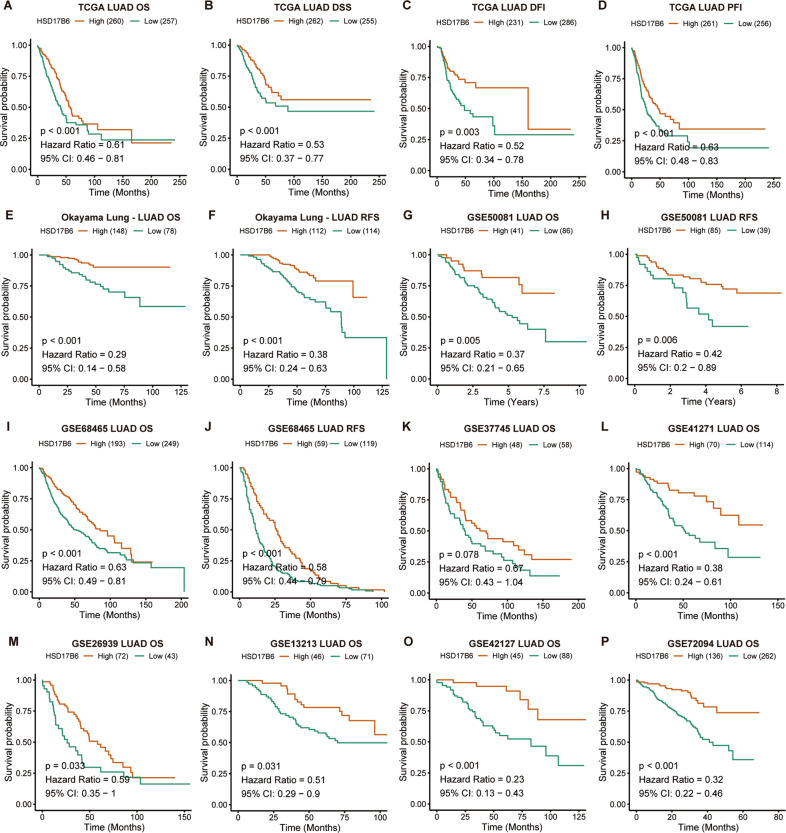
Low HSD17B6 expression is associated with poor survival in LUAD patients. **A**–**D** Kaplan–Meier analysis of OS, DSS, DFI, and PFI according to HSD17B6 expression in TCGA LUAD dataset. **E**, **F** Kaplan–Meier analysis of OS and RFS according to HSD17B6 expression in the “Okayama Lung” dataset. **G**, **H** Kaplan–Meier analysis of OS and RFS according to HSD17B6 expression in the GSE50081 LUAD dataset. **I**, **J** Kaplan–Meier analysis of OS and RFS according to HSD17B6 expression in the GSE68465 LUAD dataset. **K**–**P** Kaplan–Meier analysis of OS according to HSD17B6 expression in the GSE37745 (**K**), GSE41271 (**L**), GSE26939 (**M**), GSE13213 (**N**), GSE42127 (**O**), and GSE72094 (**P**) LUAD dataset (red, high HSD17B6 expression; green, low HSD17B6 expression). OS overall survival, DSS disease-specific survival, DFI disease-free interval, PFI progression-free interval, RFS recurrence-free survival, CI confidence interval.

Subsequently, we explored the association between HSD17B6 expression and prognosis of LUAD patients. Kaplan–Meier Plotter analysis in TCGA LUAD showed that low expression of HSD17B6 was closely correlated with shorter OS (overall survival) (*p* < 0.001), PFS (progression-free survival) (*p* < 0.001), RFS (relapse-free survival) (*p* = 0.003), and DSS (disease-specific survival) (*p* < 0.001) (Fig. 3A–D), indicating a poor prognosis in LUAD with low HSD17B6 expression. Analysis of oncomine and GEO datasets also showed that patients with LUAD expressing low levels of HSD17B6 had significantly shorter overall survival (*p* < 0.001 for “Okayama Lung”, *p* = 0.005 for GSE50081, *p* < 0.001 for GSE68465, *p* < 0.001 for GSE41271, *p* < 0.033 for GS26939, *p* = 0.031 for GSE13213, *p* < 0.001 for GSE42127, *p* < 0.001 for GSE72094, respectively) (Fig. 3E, G, I, K–P). And shorter RFS was also observed in patients with LUAD expressing low levels of HSD17B6 in lung cancer datasets of oncomine and GEO datasets (*p* < 0.001 for “Okayama Lung”, *p* = 0.006 for GSE50081, *p* < 0.001 for GSE68465, respectively) (Fig. 3F, H, J).

Based on the above results, these initial results suggest that HSD17B6 is a positive LUAD prognostic factor and maybe a new tumor suppressor of LUAD.

### Regulation of HSD17B6 expression in lung adenocarcinoma

We then sought to define the possible genetic/epigenetic mechanisms leading to HSD17B6 loss in LUAD. Promoter DNA methylation is a vital epigenetic mechanism to inhibit gene expression, and our previous study showed that it is the major contributor in regulating HSD17B6 expression in hepatocellular carcinoma [12]. However, we found that the methylation level of HSD17B6 promoter in LUAD was very low by analyzing the methylation data from TCGA LUAD (Fig. S2C). In addition, BSP analysis of CpG island upstream of HSD17B6 in lung cancer cell lines (H1299 and H1975) also showed its hypomethylation status (Fig. S2A, B). On the other hand, no positive association between HSD17B6 mRNA expression and its copy number was observed in TCGA LUAD and “Chen et al. Nat Genet, 2020” LUAD dataset [14] (Fig. S2D, E). Thus, these data demonstrate that neither promoter methylation nor CNV (copy number variation) is the major regulator of HSD17B6 expression in LUAD.

**Fig. 4:**
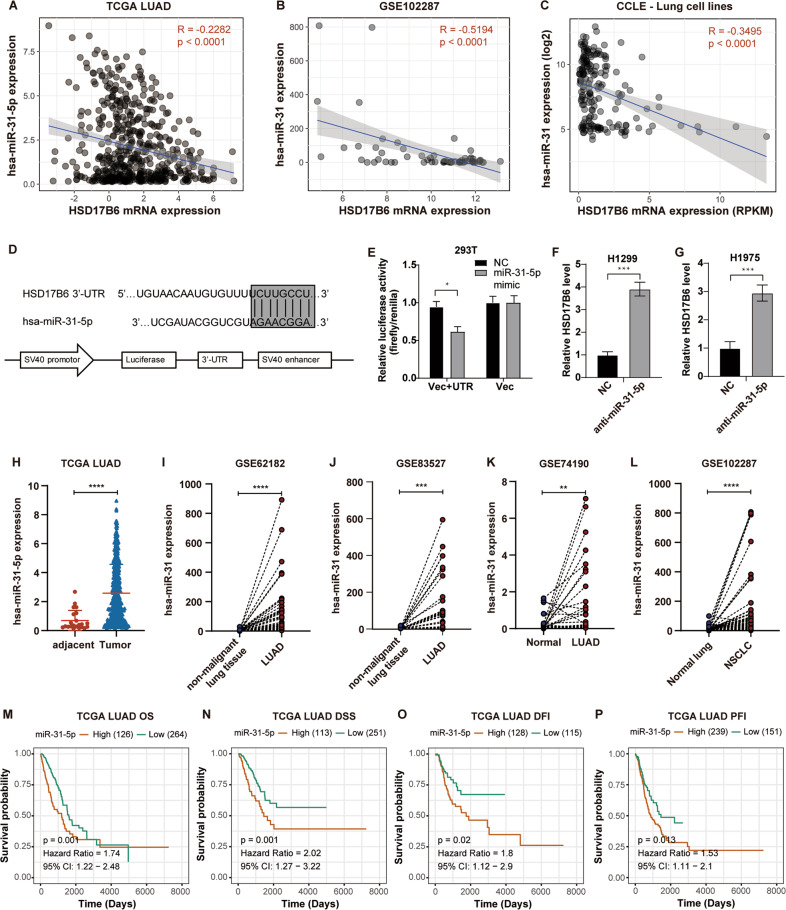
HSD17B6 expression is regulated by miR-31-5p. **A** Correlation analysis between HSD17B6 mRNA expression and miR-31-5p expression in TCGA LUAD. **B**, **C** The correlation between HSD17B6 mRNA expression and miR-31 expression in GSE102287 dataset and CCLE lung cell lines. **D** A reporter plasmid with the 3′-UTR of HSD17B6 gene targeted by miR-31-5p was constructed by inserting the UTR region downstream of the luciferase gene. **E** The relative luciferase activity (fold) of the vector with the HSD17B6-UTR sequence relative to the control vector (Vec, without UTR sequence) was determined in 293T cells transfected with the miR-31-5p mimic or scramble negative control (NC). The tests were repeated three times. Data were expressed as mean ± SD. **F**, **G** The effect of miR-31-5p inhibitor on the mRNA expression of HSD17B6 in H1299 and H1975 cells. The tests were repeated three times. Data were expressed as mean ± SD. **H** Comparison of miR-31-5p expression levels between adjacent normal tissues and LUAD tissues in TCGA LUAD. **I**–**L** Comparison of miR-31 expression levels between LUAD and paired non-cancerous samples in GSE62182, GSE83527, GSE74190, and GSE102287 datasets. **M**–**P** Kaplan–Meier analysis of OS, DSS, DFI, and PFI according to miR31-5p expression in the TCGA LUAD (red, high miR31-5p expression; green, low miR31-5p expression). OS overall survival, DSS disease-specific survival, DFI disease-free interval, PFI progression-free interval, RFS recurrence-free survival. ****p < 0.0001, ***p < 0.001, **p < 0.01, *p < 0.05.

It is well known that miRNA could negatively modulate gene expression by targeting the 3′-UTR of targeted mRNA. There were 27 miRNAs predicted to target HSD17B6 based on the overlapped prediction results of miRDB (http://mirdb.org/miRDB/) and TargetScan (http://www.targetscan.org/vert_72/) (data not shown). Among these miRNAs, miR-31-5p has the second-highest target score in miRDB. It is the only miRNA whose expression negatively correlates with HSD17B6 expression significantly in TCGA LUAD (*R* = −0.2282, *p* < 0.0001, Fig. 4A). Analysis from lung cancer dataset GSE112087 and CCLE lung cancer cell lines also confirmed the negative correlation (Fig. 4B, C). Furthermore, the miR-31-5p level was higher in H1975 than in H1299, while the mRNA level of HSD17B6 was lower in H1975 than in H1299, as shown by quantitative RT-PCR assays (Fig. S3A). To validate the direct binding of HSD17B6 and miR-31-5p, 3′-UTR of HSD17B6 (449 bp) was cloned into pGL3-control vector at the downstream of the firefly luciferase gene, creating a pGL3-HSD17B6 UTR construct (Fig. 4D). The pGL3-HSD17B6 UTR and pGL3 control were individually transfected into 293T cells. The luciferase activity of pGL3-HSD17B6 UTR but not pGL3 decreased by 45% in cells transfected with miR-31-5p mimic (Fig. 4E). We further examined the HSD17B6 level in miR-31-5p inhibitor transfected H1299 and H1975 cells versus the NC (negative control) transfected cells. It showed that miR-31-5p inhibitor transfection increased the mRNA level of HSD17B6 by about 3–4-folds in H1299 and H1975 cells (Fig. 4F, G). Furthermore, miR-31-5p expression in LUAD was significantly higher than in adjacent non-cancerous lung tissues through the analysis from TCGA LUAD and four GEO lung cancer datasets (Fig. 4H, L). Moreover, Kaplan–Meier Plotter analysis showed that high miR-31-5p expression was correlated with shorter OS (*p* = 0.001), DSS (*p* = 0.001), DFI (*p* = 0.02), and PFI (*p* = 0.013) in TCGA LUAD (Fig. 4M–P), indicating a poor prognosis in patients with LUAD expressing a high level of miR-31-5p.

Altogether, these results suggest that low HSD17B6 expression in LUAD would result from high expression of miR-31-5p in LUAD. Furthermore, high miR-31-5p expression predicts poor prognosis in patients with LUAD.

### HSD17B6 suppresses progression of lung adenocarcinoma

To determine potential function of HSD17B6 in LUAD development, we used GSEA (Gene Set Enrichment Analysis) to look for HSD17B6-associated HALLMARKs. Analyses were performed in three LUAD datasets: TCGA LUAD, GSE68465, and GSE72094. Each dataset contains more than 400 LUAD samples. The common HSD17B6-associated HALLMARKs in all three datasets were used for further analysis. Seven HALLMARKs, such as “P53 PATHWAY”, were positively associated with HSD17B6 expression, while thirteen HALLMARKs, such as “G2M CHECKPOINT”, “MITOTIC SPINDLE”, “DNA REPAIR”, “PI3K AKT MTOR SIGNALING”, and “MTORC1 SIGNALING”, were negatively associated with HSD17B6 expression (Table S2).

**Fig. 5:**
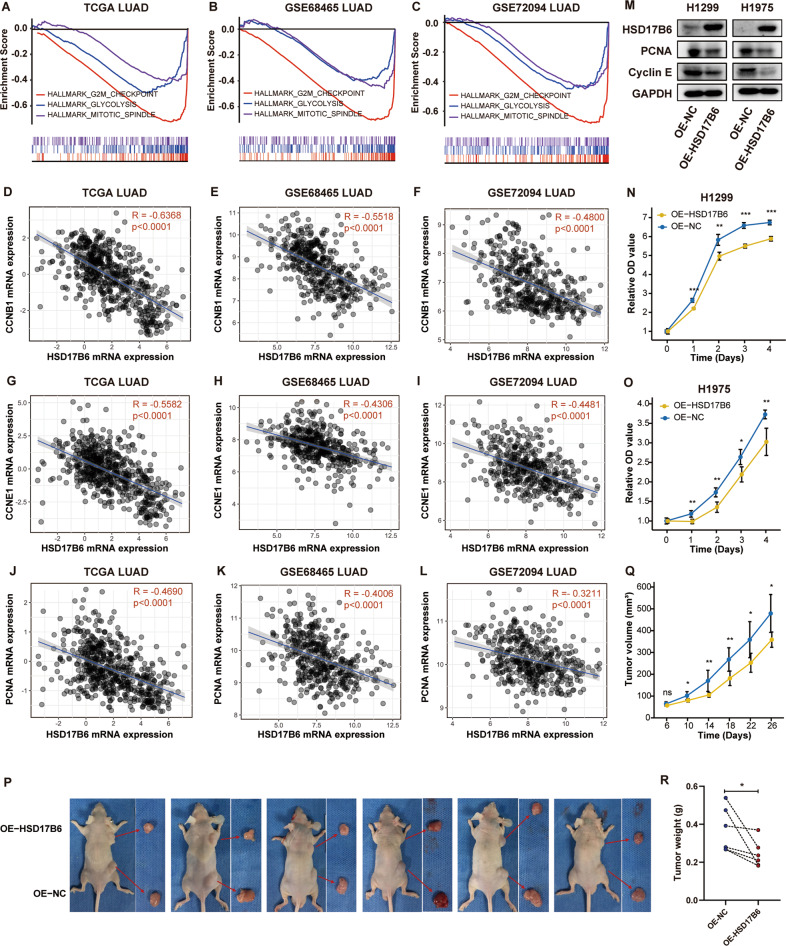
HSD17B6 impedes proliferation of LUAD cells. **A**–**C** GSEA analysis in TCGA LUAD, GSE68465, and GSE72094 LUAD showed that HSD17B6 expression levels were negatively correlated with gene signatures of “HALLMARK GLYCOLYSIS”, “HALLMARK G2M CHECKPOINT”, and “HALLMARK MITOTIC SPINDLE”. **D**–**F** Correlation of HSD17B6 mRNA expression with CCNB1 mRNA expression in TCGA LUAD, GSE68465, and GSE72094 LUAD. **G**–**I** Correlation of HSD17B6 mRNA expression with CCNE1 mRNA expression in TCGA LUAD, GSE68465, and GSE72094 LUAD. **J**–**L** Correlation of HSD17B6 mRNA expression with PCNA mRNA expression in TCGA LUAD, GSE68465, and GSE72094 LUAD. **M** Protein levels of HSD17B6, Cyclin E, and PCNA were assessed by western blotting after HSD17B6 overexpression. **N**, **O** The effect of HSD17B6 overexpression on cell proliferation of H1299 and H1975, as determined using the CCK-8 assay. **P** Six nude mice carrying subcutaneous tumors of HSD17B6-overexpressing H1299 group (top) and control group (bottom) were shown. **Q** Tumor diameter in six mice was measured every 4 days. The tumor volume was calculated and tumor growth curves were plotted over time. **R** Tumor weights were measured and compared between two groups. OE-NC: negative control for overexpression; OE-HSD17B6: HSD17B6 overexpression. ***p < 0.001, **p < 0.01, *p < 0.05.

Cell cycle and proliferation-related HALLMARKs, including “G2M CHECKPOINT”, “MITOTIC SPINDLE”, and “GLYCOLYSIS”, were significantly negatively associated with HSD17B6 expression in all three LUAD datasets (Table S2, Fig. 5A–C). In addition, correlation analysis showed that the mRNA levels of mitosis marker genes, such as PCNA, CCNB1 (cyclin B1), and CCNE1 (cyclin E1), were negatively correlated with HSD17B6 expression in TCGA LUAD (*R* = −0.6368, *R* = −0.5582, *R* = −0.4690, *p* < 0.0001, respectively), which was confirmed in GSE68465 and GSE72094 datasets (Fig. 5D–L). And the protein expressions of Cyclin B and PCNA were also correlated positively with HSD17B6 protein expression in CPTAC LUAD (cyclin E protein expression is not available in CPTAC LUAD) (Fig. S4A, B). These results suggested increased cell proliferation in LUAD with low HSD17B6 expression. Therefore, we hypothesize that HSD17B6 inhibits proliferation in LUAD. To investigate the function of HSD17B6 in LUAD, HSD17B6 gene expression in H1299 and H1975 was overexpressed, and the efficiency was detected by Western blot assay (Fig. 5M). The Cyclin E and PCNA expression strongly decreased in HSD17B6-overexpressing cells (Fig. 5M). We evaluated the effect of HSD17B6 on the proliferation of LUAD cells using a CCK-8 assay and found that HSD17B6 overexpression inhibited cell proliferation significantly in both H1299 and H1975 cell lines (Fig. 5N, O). In addition, miR-31-5p inhibitor also hindered their proliferation (Fig. S3B, C). Then, we inoculated H1299 cells overexpressing HSD17B6 or negative control into nude mice. Twenty-six days after the injection, the tumors formed in the HSD17B6-overexpressing group were substantially smaller than those in the control group (Fig. 5P, Q). Moreover, the tumor weight at the end of the experiment was also markedly lower in the HSD17B6-overexpressing group than in control group (Fig. 5R). Thus, HSD17B6 could inhibit LUAD cell proliferation both in vitro and in vivo.

**Fig. 6:**
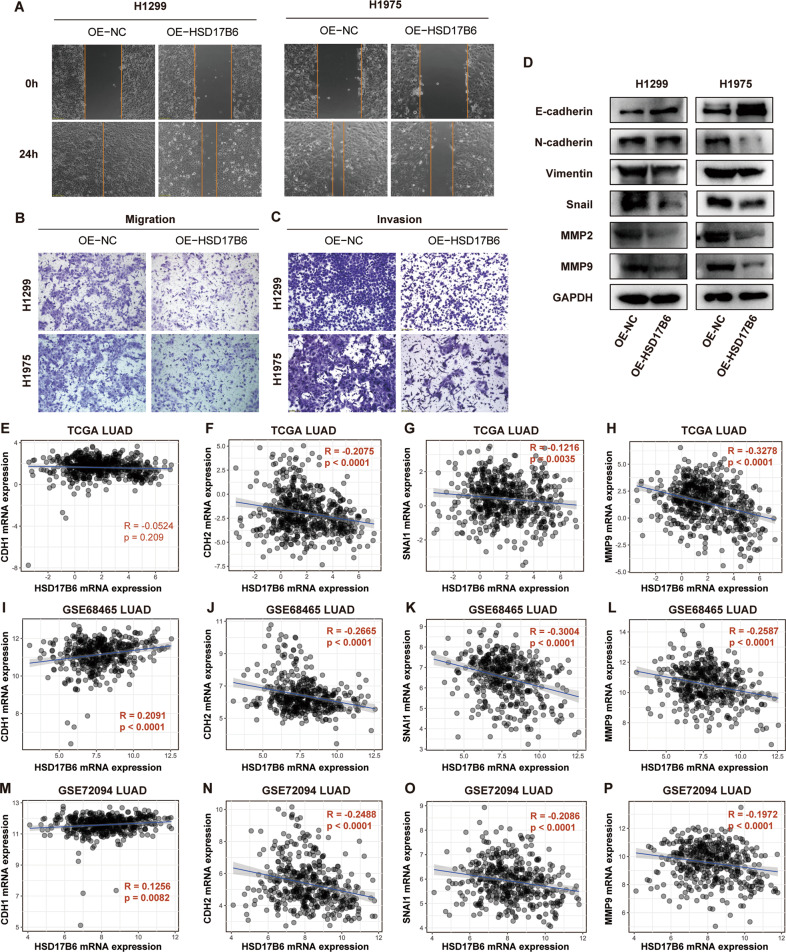
HSD17B6 inhibits the cell migration and invasion of LUAD cells. **A** The effect of HSD17B6 overexpression on cell migration of H1299 and H1975, as determined using wound-healing assays. **B, C** The effect of HSD17B6 on cell migration and invasion of H1299 and H1975, as determined using transwell assay. **D** Protein levels of E-cadherin, N-cadherin, Vimentin, Snail, MMP2, and MMP9 were assessed by western blotting after HSD17B6 overexpression. OE-NC, negative control for overexpression; OE-HSD17B6, HSD17B6 overexpression. **E–P** Correlation analysis of HSD17B6 expression and expression of invasion-related genes (CDH1, CDH2, SNAI1, and MMP9) in TCGA LUAD, GSE68465, and GSE72094 datasets.

As low HSD17B6 expression levels were associated with advanced N/M stages (Fig. 2S1), we hypothesized that HSD17B6 might inhibit invasion in LUAD. HSD17B6 overexpression in H1299 and H1975 cells led to a significant reduction in cell migration as demonstrated by wound-healing assay and transwell assay (Fig. 6A, B). HSD17B6 also substantially inhibited the invasion of H1299 and H1975 cells, as demonstrated by Matrigel-coated transwell assay (Fig. 6C). In addition, miR-31-5p inhibitor also attenuated the migration and invasion of H1299 and H1975 cells as demonstrated with wound-healing assay and transwell assay (Fig. S3D, E). EMT (epithelial–mesenchymal transition) is essential for the pathogenesis of metastasis and tumor cell migration [15]. During the EMT, cells lose their epithelial markers and acquire mesenchymal features. Western blot analysis showed that HSD17B6 overexpression enhanced the expression of E-cadherin (epithelial marker) while decreased the expression of vimentin and N-cadherin (mesenchymal markers) (Fig. 6D), indicating that HSD17B6 inhibited the EMT in LUAD cells. Consistent with this, examining the mRNA level of genes from the above three LUAD datasets showed that HSD17B6 mRNA expression was positively correlated with CDH1 (encoding E-cadherin) while negatively correlated with CDH2 (encoding N-cadherin) (Fig. 6E, F, I, J, M, and N). Furthermore, analyzing the protein level of these genes from CPTAC LUAD also showed that HSD17B6 protein expression was positively correlated with E-cadherin while negatively correlated with N-cadherin (Fig. S4C, D). Furthermore, HSD17B6 significantly increased the protein levels of Snail, which is a key EMT-promoting transcriptional factor (Fig. 6D). Consistently, the mRNA levels of SNAI1 (encoding Snail) were negatively correlated with mRNA levels of HSD17B6 (Fig. 6G, K, and O). These results demonstrated that HSD17B6 repressed EMT process in LUAD. In addition, HSD17B6 overexpression suppressed the expression of MMP2 and MMP9, which are critical proteinases for tumor invasion and metastasis through degrading extracellular matrix and vascular basement membrane [16]. Accordantly, there was a significant inverse correlation between HSD17B6 and MMP9 mRNA expression in TCGA LUAD, GSE68465 LUAD, and GSE72094 LUAD datasets (Fig. 6H, L, and P). In addition, MMP9 protein expression was inversely correlated with HSD17B6 protein expression in CPTAC LUAD dataset (Fig. S4E).

**Fig. 7:**
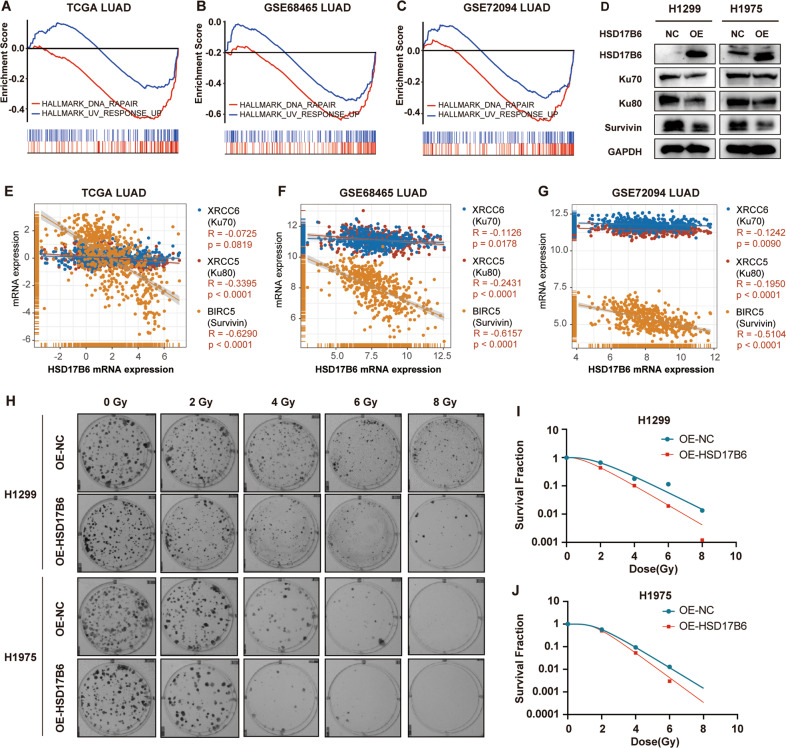
HSD17B6 reduces radioresistance in lung cancer cells. **A**–**C** GSEA analysis revealed that HSD17B6 expression levels were negatively correlated with gene signatures of “HALLMARK DNA REPAIR” and “HALLMARK UV RESPONSE UP” in TCGA LUAD, GSE68465, and GSE72094 datasets. **D** Protein levels of Ku70, Ku80, and Survivin were assessed by western blotting after HSD17B6 overexpression. **E**–**G** Correlation analysis of HSD17B6 expression and expression of DNA repair/survival-related genes (XRCC6, XRCC5, and BIRC5) in TCGA LUAD, GSE68465, and GSE72094 datasets. **H** Clonogenic survival of H1299 and H1975 following radiation. **I** Cell survival curves of H1299 OE-NC (blue line) and H1299 OE-HSD17B6 cells (red line) were plotted by the multi-target single-hit model. **J** Cell survival curves of H1975 OE-NC (blue line) and H1975 OE-HSD17B6 cells (red line) were plotted by the multi-target single-hit model.

DNA damage/repair-related HALLMARKs, including “DNA REPAIR” and “UV RESPONSE UP”, was significantly negatively associated with HSD17B6 expression in all three LUAD datasets (Table S2, Fig. 7A–C). DNA damage repair of DSBs is error-prone and increases gene mutations and genome instability, promoting tumor progression and worsening tumor prognosis of tumor [17]. Analysis in TCGA LUAD showed that mutation count, MSI (Microsatellite instability) score, and aneuploidy (Chromosomal instability) score were correlated negatively with HSD17B6 expression (Fig. S5). Radiotherapy, which kills tumor cells mainly by DNA damage, is an important therapeutic strategy against lung cancer, and weakened DNA damage repair capacity promotes radiosensitivity. HSD17B6 overexpression suppressed Ku70, Ku80, and survivin expression, which are crucial proteins involved in DNA repair and anti-apoptosis (Fig. 7D). Furthermore, correlation analysis revealed that HSD17B6 mRNA expression correlated inversely with mRNA expression of XRCC6, XRCC5, and BIRC5 in LUAD datasets, which encode Ku70, Ku80, and Survivin, respectively (Fig. 7E–G). Next, we analyzed the effect of HSD17B6 on clonogenic survival ability after irradiation. HSD17B6 overexpression in H1299 and H1975 cells sensitized these cells to radiation (Fig. 7H–J). In addition, miR-31-5p inhibitor also sensitized these cells to radiation (Fig. S3F–H).

Collectively, our data support that HSD17B6 could attenuate cell proliferation, EMT, migration, invasion, and radioresistance in LUAD.

### HSD17B6 inhibits LUAD progression through the PTEN-AKT pathway

**Fig. 8:**
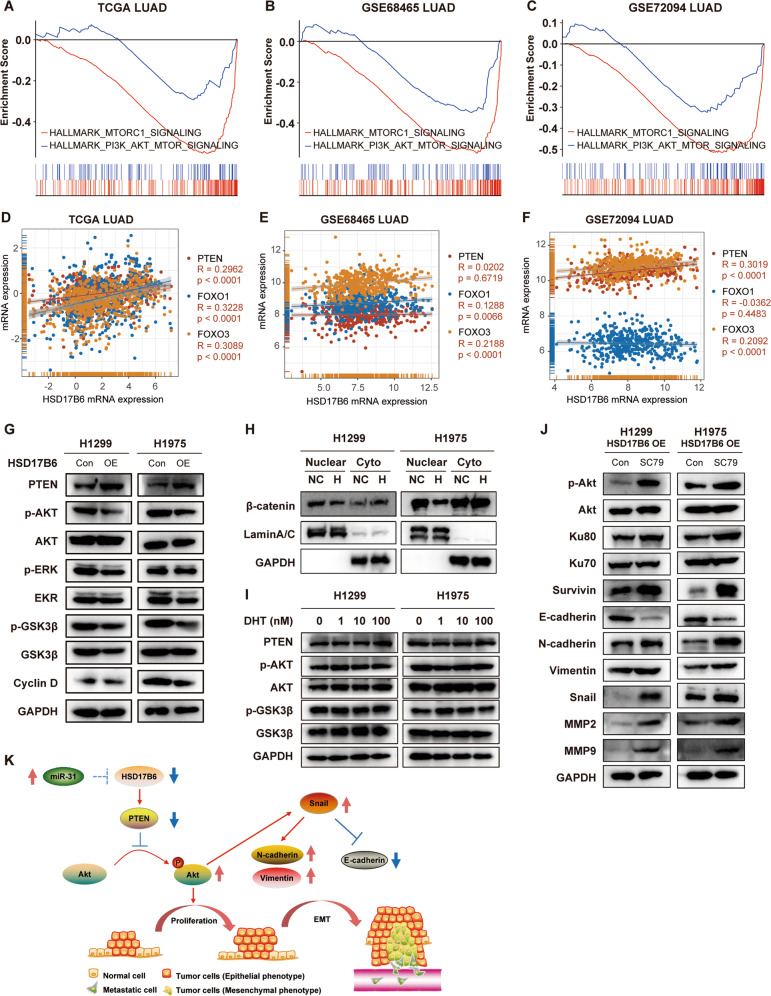
Effects of HSD17B6 on the AKT signaling pathway. **A**–**C** GSEA analysis in TCGA LUAD, GSE68465, and GSE72094 LUAD showed that HSD17B6 expression levels were negatively correlated with gene signatures of “HALLMARK PI3K AKT MTOR SIGNALING” and “HALLMARK MTORC1 SIGNALING”. **D**–**F** Correlation analysis of HSD17B6 expression and expression of genes in the Akt signaling pathway (PTEN, FOXO1, and FOXO3) in TCGA LUAD, GSE68465, and GSE72094 datasets. **G** Protein levels of PTEN, p-AKT (Ser473), Akt, p-ERK, ERK, p-GSK3β, GSK3β, and Cyclin D were assessed by western blotting after HSD17B6 overexpression. **H** Relative protein levels of nuclear and cytosolic β-catenin were assessed by western blotting after HSD17B6 overexpression. Lamin A/C served as the nuclear marker, and GAPDH served as the cytosolic marker. Cyto: cytoplasmic; NC: negative control; H: HSD17B6 overexpression. **I** Western blot analysis of protein levels in Akt signaling pathway, including PTEN, Akt, p-Akt (Ser473), GSK3β, p-GSK3β in H1299 and H1975 cells treated with increasing concentrations of DHT for 24 h. **J** Akt activator SC79 reversed the effects of HSD17B6 overexpression on the protein expression of Ku80, Ku70, Survivin, E-cadherin, N-cadherin, Vimentin, Snail, MMP2, and MMP9. **K** Schematic summarizing the results of the study.

We next addressed the molecular mechanism through which HSD17B6 regulates LUAD progression. GSEA analysis in TCGA LUAD, GSE68465 LUAD, and GSE72094 LUAD datasets showed that gene signature of “HALLMARK PI3K AKT MTOR SIGNALING” was significantly upregulated in LUAD with low HSD17B6 expression (Table S2, Fig. 8A–C). Furthermore, gene signature of “HALLMARK MTORC1 SIGNALING”, a downstream signaling pathway of PI3K/AKT, was also inversely correlated with HSD17B6 expression in these datasets (Table S2, Fig. 8A–C). PI3K/AKT signaling pathway is a prominent positive regulator of cell-cycle progression, EMT, and radioresistance [18] and is constitutively active in various cancer types, including lung cancer [19]. Therefore, we hypothesized that HSD17B6 might attenuate PI3K/AKT signaling pathway in LUAD. Western blot analysis showed that HSD17B6 inhibited Akt (Ser473) phosphorylation but did not change total Akt in H1299 and H1975 cells (Fig. 8G). In addition, downstream signaling events, including GSK-3β (Ser9) phosphorylation, cyclin D1 expression, and nuclear accumulation of β-catenin, were also suppressed by HSD17B overexpression in H1299 and H1975 cells (Fig. 8G, H). And PTEN, an upstream suppressor of pAKT, was upregulated in HSD17B6-overexpressing H1299 and H1975 cells (Fig. 8G). Besides, PTEN transcription levels were positively correlated with HSD17B6 levels in LUAD datasets. There was also a positive correlation between HSD17B6 mRNA levels and levels of FOXO1 and FOXO3 genes, both of which are known downstream negative effectors of pAkt (Fig. 8D–F) [20]. In addition, treatment with DHT, a syntenic product of HSD17B6, did not change the protein level of PTEN and p-Akt in these lung cancer cells, suggesting a DHT-independent function for HSD17B6 in LUAD tumorigenesis (Fig. 8I).

SC79 is a newly-developed Akt activator [21]. We, therefore, wanted to know if SC79 could reverse the downstream protein expression induced by HSD17B6 overexpression. After treatment with SC79 in HSD17B6-overexpressing H1299 and H1975 cells, the expression of phosphorylated Akt, Ku70, Ku80, Survivin, N-cadherin, Vimentin, Snail, MMP2, and MMP9 increased, while the expression of E-cadherin decreased (Fig. 8J).

Altogether, we conclude that HSD17B6 loss promotes LUAD progression by downregulating PTEN expression and activating the AKT signaling pathway (Fig. 8K).

## Discussion

In the present study, the tumor suppressor role of HSD17B6 in LUAD was clarified. First, using the clinical mRNA datasets from TCGA, Oncomine, GEO datasets, and protein datasets from CPTAC, we have shown that both mRNA and protein levels of HSD17B6 dramatically decreased in LUAD tissues, which correlated with obvious poorer OS, PFS, RFS, DSS and advanced T stage, N stage, M stage, grade and pathological stage in LUAD. All these results indicated that HSD17B6 might serve as a favorable prognostic biomarker for LUAD. Second, the low expression of HSD17B6 in LUAD was probably mainly caused by abnormally high expression of miR-31-5p. Third, further study revealed an inhibitory role of HSD17B6 in the regulation of proliferation, invasion, and radioresistance of LUAD cells, suggesting that HSD17B6 is a critical mediator of LUAD progression. Finally, we demonstrated a possible mechanistic link between HSD17B6 and LUAD progression through the PTEN/AKT signaling pathway.

Radiotherapy (RT) is required at least once in over half of patients in both curative and palliative treatments across all stages of lung cancer [22]. Ionizing radiation induces cell death mainly through induction of DNA double-strand breaks (DSBs), the most lethal form of DNA damage [23]. Cell survival after DNA damage depends on DNA repair, and the lack of DNA repair would lead to genomic instability and cell death. Most DSBs induced by radiation are repaired by non-homologous end joining (NHEJ) in human cells [24]. As anticipated, enhanced DNA damage repair capacity in tumor cells would lead to resistance to radiation. Here, we have demonstrated that the key components involved in NHEJ, ku70, and ku80, were downregulated in HSD17B6-overexpressing LUAD cells, accompanied by the increased sensitivity to ionizing radiation and decreased expression level of anti-apoptotic protein survivin. Therefore, loss of HSD17B6 functions in LUAD conferred tumor cell resistance against radiotherapy, thus leading to poor prognosis in patients.

EMT is the key process that drives cancer invasion and metastasis. It induces epithelial cells to multiple biochemical changes, resulting in altered morphogenesis and loss of cell–cell and cell–extracellular matrix (ECM) adhesion [25]. In the present study, we found that HSD17B6 facilitated expression of epithelial marker E-cadherin and inhibited expression of mesenchymal marker N-cadherin. The key positive regulator of EMT, Snail, was also significantly inhibited by HSD17B6. Moreover, HSD17B6 mRNA levels were positively correlated with CDH1 (E-cadherin) levels while inversely correlated with CDH2 (N-cadherin) levels in clinical LUAD specimens. These results suggested that HSD17B6 might suppress EMT both in vitro and in vivo. Mechanically, expression levels of p-Akt, p-GSK-3β, and nuclear β-catenin were decreased when HSD17B6 was overexpressed. Meanwhile, the expression of PTEN, which is a crucial upstream negative regulator of pAKT, was increased after HSD17B6 overexpression. Furthermore, SC79 (a pAKT activator) reversed the effect of HSD17B6 overexpression on expression of EMT/invasion and DNA repair/Survivin related protein. These results strongly indicated that activation of the Akt signaling pathway was responsible for the loss of HSD17B6-induced EMT and thus metastasis in LUAD. In accordance with our results, the crucial role of this pathway during EMT has also been demonstrated previously in multiple cancers, such as colorectal cancer [26], nasopharyngeal carcinoma [27], HCC [28], and NSCLC [29].

It has been reported that dysregulation of miR-31-5p promotes tumor progression in multiple cancers. For example, miR-31-5p promotes proliferation and invasion in osteosarcoma cells and colorectal cancer cells [30, 31]. And it increases chemoresistance in malignant pleural mesothelioma [32]. A recent study has demonstrated that hypoxia-derived exosomal miR-31-5p promotes LUAD metastasis [33]. In this study, we also found that miR-31-5p could promote migration and invasion of LUAD cells. In addition, we found miR-31-5p could promote proliferation and radioresistance of LUAD cells. What’s more, we found that miR-31-5p is significantly higher in LUAD tissues than in normal tissue, and its high level is related to poor prognosis of LUAD patients. Our study showed that HSD17B6 is one of its targets, and miR-31-5p might promote the LUAD progression partly by decreasing HSD17B6 expression. However, other target genes of miR-31-5p might also be involved in regulating these progressions, such as SATB2 [33].

The protein encoded by HSD17B6 could convert 3-α-Adiol to DHT [7, 8], dysregulation of which influence the progression of multiple kinds of tumor [9]. For example, high levels of DHT arrested cell-cycle progression and induced apoptosis in human liver cells [34]. However, we showed here that DHT did not affect the activity of the PTEN-Akt signaling pathway in LUAD cells, suggesting a DHT-independent function of HSD17B6 in LUAD. Thus, additional studies will be needed to elaborate on how HSD17B6 inhibits Akt activation.

In summary, low HSD17B6 is frequently observed in LUAD and is associated with advanced tumor stage, grade, and poor prognosis in LUAD, resulting from high miR-31-5p expression in LUAD. HSD17B6 inhibited tumor cell proliferation, EMT, invasion, and radioresistance through inhibiting AKT and its downstream signaling pathways. This study indicates that HSD17B6 could be a promising biomarker and therapeutic target for LUAD.

## Materials and methods

### Data acquisition in TCGA LUAD, Oncomine, CPTAC, and GEO datasets

Phenotype data, copy number (gene-level), DNA methylation, and normalized gene expression by RNAseq in TCGA LUAD were retrieved through the UCSC XENA (https://xenabrowser.net) [35]. Oncomine datasets of LUAD, including Bhattacharjee lung [36], Gaber lung [37], Hou lung [38], Landi lung [39], Okayama lung [40], Sellamat lung [41], Stearman lung [42], Ding lung [43], and Lee lung [44], were used in this study. The HSD17B6 expression value and sample information were exported from The Oncomine platform (http://www.oncomine.com). The protein expression profiles of LUAD (CPTAC LUAD) were generated by the Clinical Proteomic Tumor Analysis Consortium (NCI/NIH) and downloaded from Linkedomics (http://www.linkedomics.org) [45, 46]. CPTAC LUAD dataset was utilized to compare the differential protein levels of HSD17B6 between tumor and normal tissues and analyze the association between HSD17B6 protein level and clinicopathological features in LUAD. Thirteen GEO (Gene Expression Omnibus) datasets of LUAD are used in this paper, including GSE50081 [47], GSE68465 [48], GSE37745 [49], GSE41271 [50], GSE26939 [51], GSE13213 [52], GSE42127 [53], GSE72094 [54], GSE102287 [55], GSE62182 [56], GSE83537 [57], GSE74190 [58], and GSE102287 [55]. The normalized expression matrix and sample information of these datasets were obtained from GEO platform (https://www.ncbi.nlm.nih.gov/geo/).

Patient survival analyses were performed using “survminer” and “survival” packages in R. Optimal cutoff value with a minimal p-value was used to dichotomize patients into low and high expression groups.

### Cell culture, lentivirus transfection, and miRNA transfection

H1299 and H1975 cells were purchased from COBIOER BIOSCIENCES (Nanjing, Jiangsu, China). They were cultured in RPMI-1640 medium supplemented with 10% FBS at 37 ˚C, 5% CO_2_. The lentivirus expressing empty lenti-vector alone and HSD17B6 were purchased from HanBio (Shanghai, China). The cells were infected with lentivirus supplemented with 5 μg/ml polybrene for 24 h, then selected with puromycin (2 μg/ml) for 14 days. The miR-31-5p mimic, miR-31-5p inhibitor, scramble sequence (negative control, NC), and the riboFECT CP transfection kit were supplied by Ribobio (Guangzhou, Guangdong, China). Transfection was performed according to the manufacturer’s instruction.

### Quantitative PCR (qRT–PCR)

The assays were performed as we have published previously [12, 59]. The sequences of the primers were listed as follows (5′→3′): HSD17B6 forward, CTCCAGCATTCTGGGAAGAG, and reverse, AAGAAGCCCCCAAGCATATT; GAPDH forward, GGAGCGAGATCCCTCCAAAAT, and reverse, GGCTGTTGTCATACTTCTCATGG. All the experiments were performed in triplicate.

### Invasion and migration assays

For the migration assay, cells were placed in inserts (8.0-μm pores, 5 × 10^4^ cells/insert). These inserts were put in 24-well plates with 10% FBS-containing media. For the invasion assay, Millicell inserts were pre-coated with 1 mg/ml of Matrigel (BD Biosciences, San Jose, CA). After 24 h of incubation, cells on the upper surface of the membrane were removed. Migrated/invaded cells on the lower surface of the membrane were fixed in methanol and then stained with crystal violet. For the wound-healing assay, cells were plated in a 24-well plate overnight. Then, the confluent monolayers were scratched with a 10-µl pipette tip. Photos of wound healing by migrating cells were taken at 0 and 24 h with a microscope (Olympus IX73, Japan). All the experiments were performed in triplicate.

### Cell proliferation assay

The cells were seeded into 96-well plates at the density of 2000 cells/well, and proliferation was measured every 24 h for 4 days. At the indicated intervals, 10 μl CCK-8 (APExBIO #K1018) was added to each well and incubated for 2 h at 37 °C, and signal absorbance at 450 nm was measured with a microplate reader (Tecan Group Ltd., Männedorf, Switzerland). All assays were repeated five replicates.

### In vivo tumor formation assay

Six 4-week-old BALB/c nude mice were obtained from the Experimental Animal Center of University of Science and Technology of China. A total of 1.5 × 10^6^ H1299 cells were inoculated subcutaneously injected into each site on the flanks (two inoculation sites per mouse, up: HSD17B6-overexpressing group, lower: negative control group). Tumor was measured every 4 days, and tumor volumes were calculated using the equation: volume = (length × width^2^)/2. At 26 days post-injection, the mice were euthanized and each tumor was weighed.

### Clonogenic survival assay for radiation

LUAD cells in the exponential growth phase were seeded at a density of 200, 400, 1000, 2000, 4000 cells/well in six-well plates and exposed to a 6 MV X‑ray in CX‑SN5340 (VARIAN) at 0 Gy, 2 Gy, 4 Gy, 6 Gy, and 8 Gy with an average dose rate of 300 cGy/min. Two weeks later, the colonies were fixed in methanol and stained with crystal violet. The colonies containing >50 cells were counted. The surviving fraction was calculated as previously described [60].

### Western blotting

For preparing cell lysates for western blotting, cells were lysed in ice-cold cell lysis buffer and centrifuged to remove cell debris. Nuclear and cytoplasmic fractions were extracted from the cell pellets as previously described [61]. After electrophoresis, proteins were transferred to a PVDF membrane. After blocking, membranes were incubated with the following primary antibodies: GAPDH (ProteinTech #60004-1-Ig), HSD17B6 (ProteinTech #11855-1-AP), AKT (CST #C6717), p-AKT (Ser473) (CST #4060), PTEN (ProteinTech #22034-1-AP), MMP2 (ProteinTech #10373-2-AP), MMP9 (ProteinTech #10375-2-AP), E-cadherin (ProteinTech #20874-1-AP), N-cadherin (ProteinTech #66219-1-Ig), Vimentin (ProteinTech #10366-1-AP), snail (CST #3895), survivin (CST #2808), GSK3β (ProteinTech #22104-1-AP), p-GSK3β (CST #9322), β-catenin (CST #8480), cyclin D1 (ProteinTech #60186-1-Ig), cyclin E1 (ProteinTech #11554-1-AP), PCNA (ProteinTech #10205-2-AP), and Lamin A/C (ProteinTech #10298-1-AP) overnight at 4 ˚C. Then, the membranes were incubated with secondary antibodies: HRP-conjugated anti‑rabbit IgG (ProteinTech #SA00001‑2) or anti‑mouse IgG (ProteinTech #SA00001‑1) for 1 h at room temperature. All the experiments were performed in triplicate.

### BSP analysis

BSP analysis was performed as previously described [62]. Briefly, genomic DNA was extracted from cells, then qualified and quantified by a NanoPhotometer (IMPLEN). The bisulfite conversion was carried out using EZ DNA Methylation-Gold Kit (cat. no. D5006; ZYMO Research). Upstream CpG Island of HSD17B6 gene was amplified using the primers listed below: forward, 5′-GATAGTATTGAGAGTAGGGAAAGAG-3′ and reverse, 5′-TTCTACCCACAAAAACRATAAC-3′. The PCR products from bisulfite-treated DNA were cloned into T- vector and then sequenced.

### Luciferase reporter assay

A full length of the human HSD17B6 3′-untranslated region (449 bp) with the miR-31-5p targeting sequence was cloned downstream of the firefly luciferase gene in pGL3-control (Invitrogen) to construct pGL3-luc-HSD17B6. Then, the luciferase activity was determined as previously described [63].

### Gene set enrichment analysis (GSEA)

Spearman’s correlation coefficient between the mRNA levels of each gene and HSD17B6 levels was computed and used to create a ranked gene list, which was supplied to pre-ranked analysis on HALLMARK-term database (h.all.v7.3.symbols.gmt) of Molecular Signatures Database (MSigDB) using GSEA software (v4.1.0). Statistically significant pathways were screened based on the <0.25, and |enriched score| >0.35 as the cutoff criteria [64].

### Statistical analysis

Data are presented as mean ± standard deviation. Differences between the two groups were evaluated using the two-sided Student’s *t*-test for normally distributed data or Mann–Whitney test for non-normally distributed data. F-test was used to compare the variance of two samples before Student’s *t*-test. Normality of data was determined by Kolmogorov–Smirnov test. Correlation analysis was performed using the Pearson’s test. Statistical analysis was performed with packages in R software or Prism 8.3.4 (GraphPad). ****p < 0.0001, ***p < 0.001, **p < 0.01, *p < 0.05. All data were analyzed blindly.

## Supplementary information

Supplementary Figure Legends

Figure S1

Figure S2

Figure S3

Figure S4

Figure S5

Table S1

Table S2

## Data Availability

The data that support the findings of the current study are available from the corresponding author on reasonable request.
