# Supplementary material for: HSD17B6 downregulation predicts poor prognosis and drives tumor progression via activating Akt signaling pathway in lung adenocarcinoma
Source: Cell Death Discov. 2021 Nov 8;7:341. doi: 10.1038/s41420-021-00737-0 (PMC8576029; doi:10.1038/s41420-021-00737-0)
Supplement: Supplementary file 1 — Supplementary Figure Legends [file 41420_2021_737_MOESM1_ESM.docx]

**Figure S1. HSD17B6 Protein levels decrease with LUAD progression.** (A) HSD17B6 protein levels were downregulated in LUAD samples compared with their paired adjacent non-neoplastic in CPTAC LUAD dataset. (B–F) HSD17B6 protein levels in different T stages (B), N stages (C), M stages (D), Grades (E), and tumor size (F) of LUAD samples in CPTAC LUAD dataset. ****p < 0.0001, **p < 0.01, *p < 0.05.

**Figure S2. HSD17B6 Expression is not regulated by DNA methylation or copy number variation (CNV)**. (A) UCSC genome browser shows the location of HSD17B6 gene and its upstream region. CpG island containing 35 CpG sites analyzed by BSP (Bisulfite sequencing PCR) in the promoter of HSD17B6 promoter region are indicated. (B) The methylation status of CpG sites in the HSD17B6 promoter was measured by BSP in H1975 and H1299 cells. The methylated CpG sites are represented with filled circles, and the unmethylated CpG sites are represented with hollow circles. (C) Heatmap of HSD17B6 mRNA expression (I) and β-value (methylation level) of 9 CpG sites (II) in HSD17B6 promoter. Samples were ordered from top to bottom by the HSD17B6 expression. Each row indicates one sample. Blue: low level; Red: high level. (D) The correlation mRNA expression and copy number of HSD17B6 in TCGA LUAD. (E) HSD17B6 mRNA expression levels in LUAD with different copy number variation from “Chen et al. Nat Genet. 2020” dataset. * P < 0.05.

**Figure S3. Effects of miR-31-5p on LUAD cells.** (A) Comparison of miR-31-5p and HSD17B6 mRNA level between H1299 and H1975 using qRT-PCR. (B-C) The effect of miR-31-5p inhibitor on cell proliferation of H1299 and H1975, as determined using the CCK-8 assay. (D) The effect of miR-31-5p inhibitor on cell migration of H1299 and H1975, as determined using the wound-healing assay. (D-E) The effect of miR-31-5p inhibitor on cell migration and invasion of H1299 and H1975, as determined using transwell assay. (F) Clonogenic survival of cells following radiation. (G-H) Cell survival curves of NC (blue line) and miR-31-5p inhibitor (red line) transfected H1299 cells (G) and H1975 cells (H) were plotted by the multi-target single-hit model.

**Figure S4.** Correlation of HSD17B6 protein expression with protein expression of Cyclin B, PCNA, E-cadherin, N-cadherin, and MMP9 in CPTAC LUAD.

**Figure S5.** Correlation of HSD17B6 mRNA expression with mutation count, aneuploidy score, and MSIsensor score in TCGA LUAD.
