# Supplementary figures and images for: HSD17B6 downregulation predicts poor prognosis and drives tumor progression via activating Akt signaling pathway in lung adenocarcinoma
Source: Cell Death Discov. 2021 Nov 8;7:341. doi: 10.1038/s41420-021-00737-0 (PMC8576029; doi:10.1038/s41420-021-00737-0)

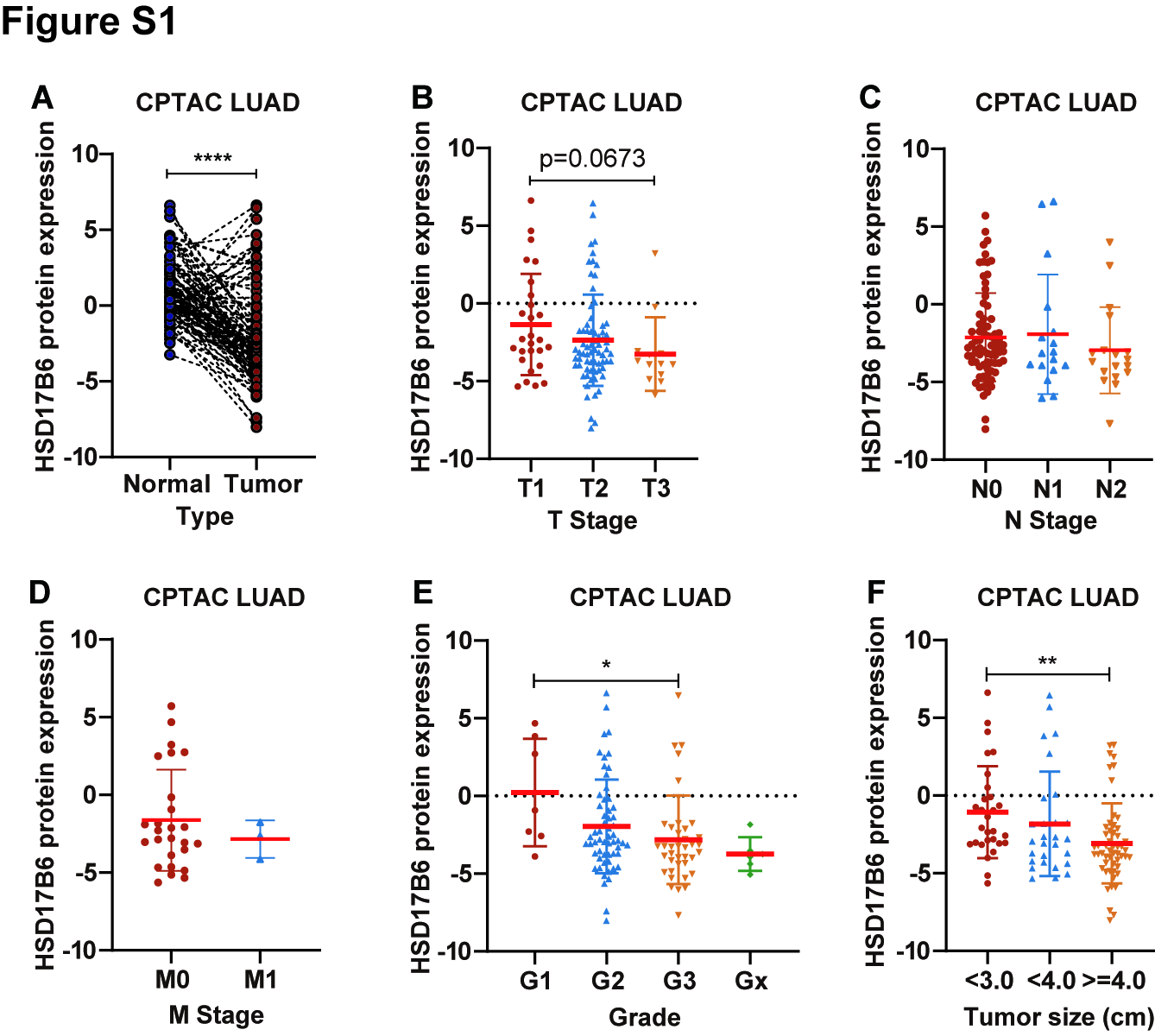

Supplement: Supplementary file 2 — Figure S1 [file 41420_2021_737_MOESM2_ESM.tif]

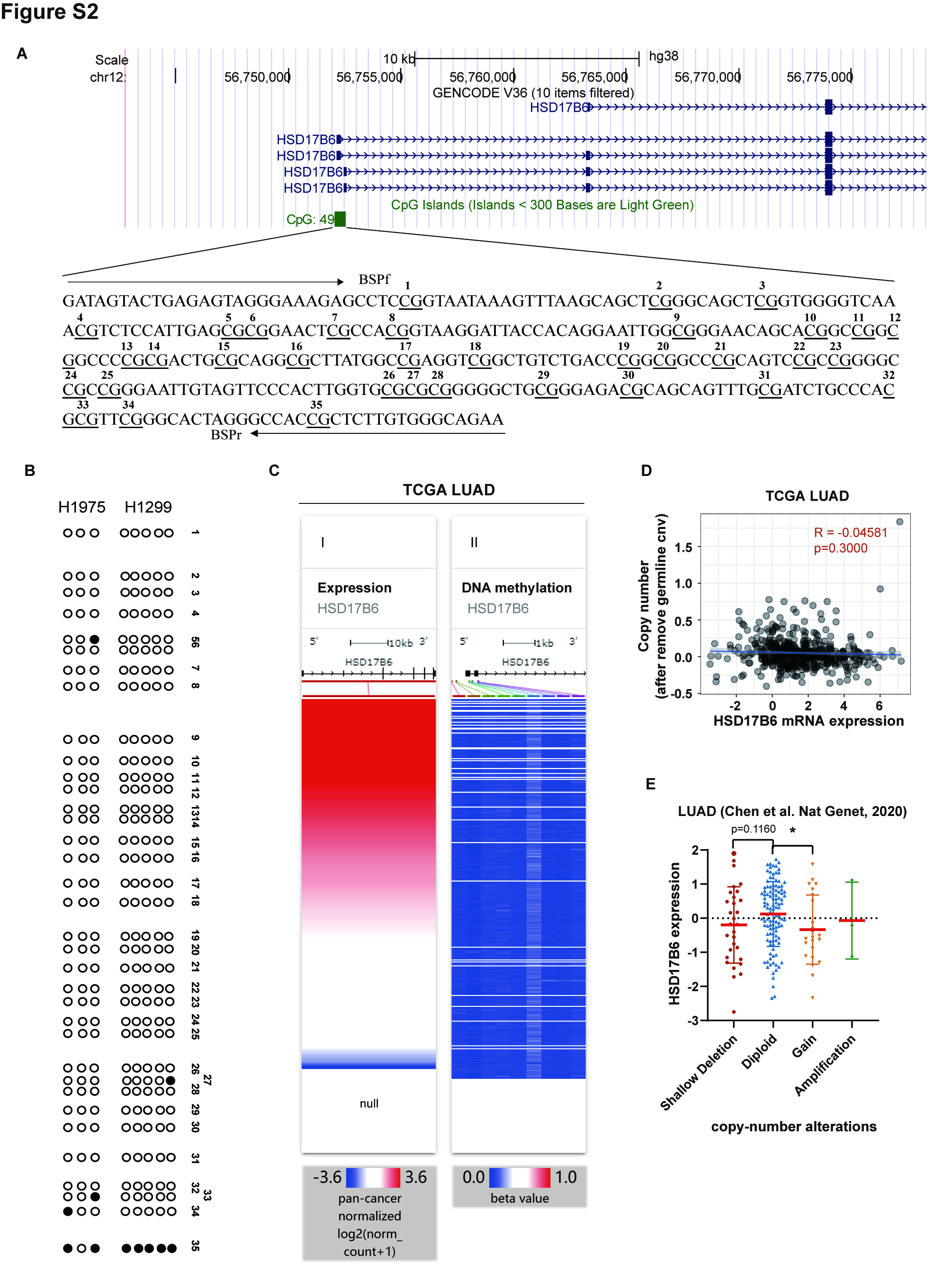

Supplement: Supplementary file 3 — Figure S2 [file 41420_2021_737_MOESM3_ESM.tif]

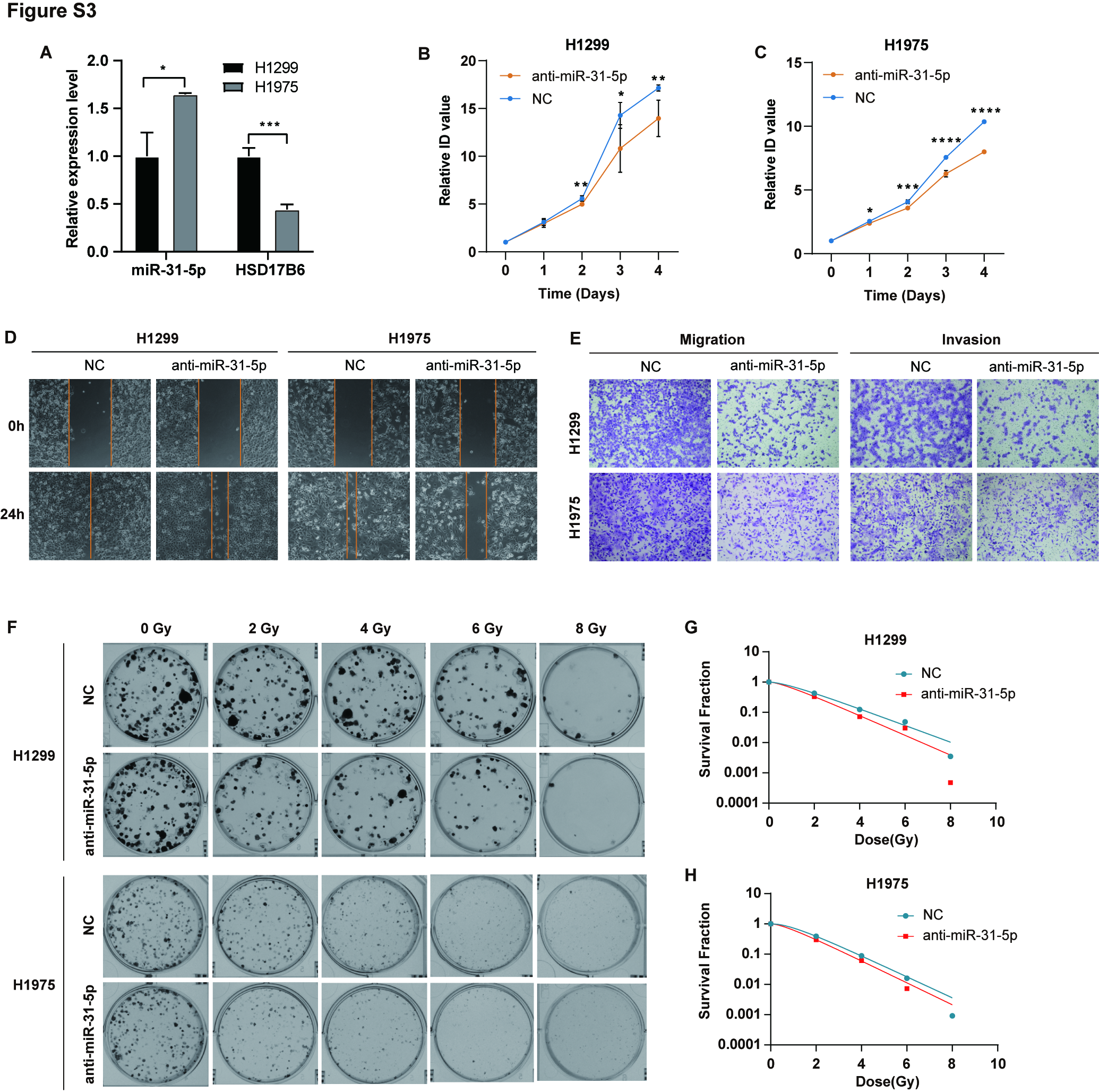

Supplement: Supplementary file 4 — Figure S3 [file 41420_2021_737_MOESM4_ESM.tif]

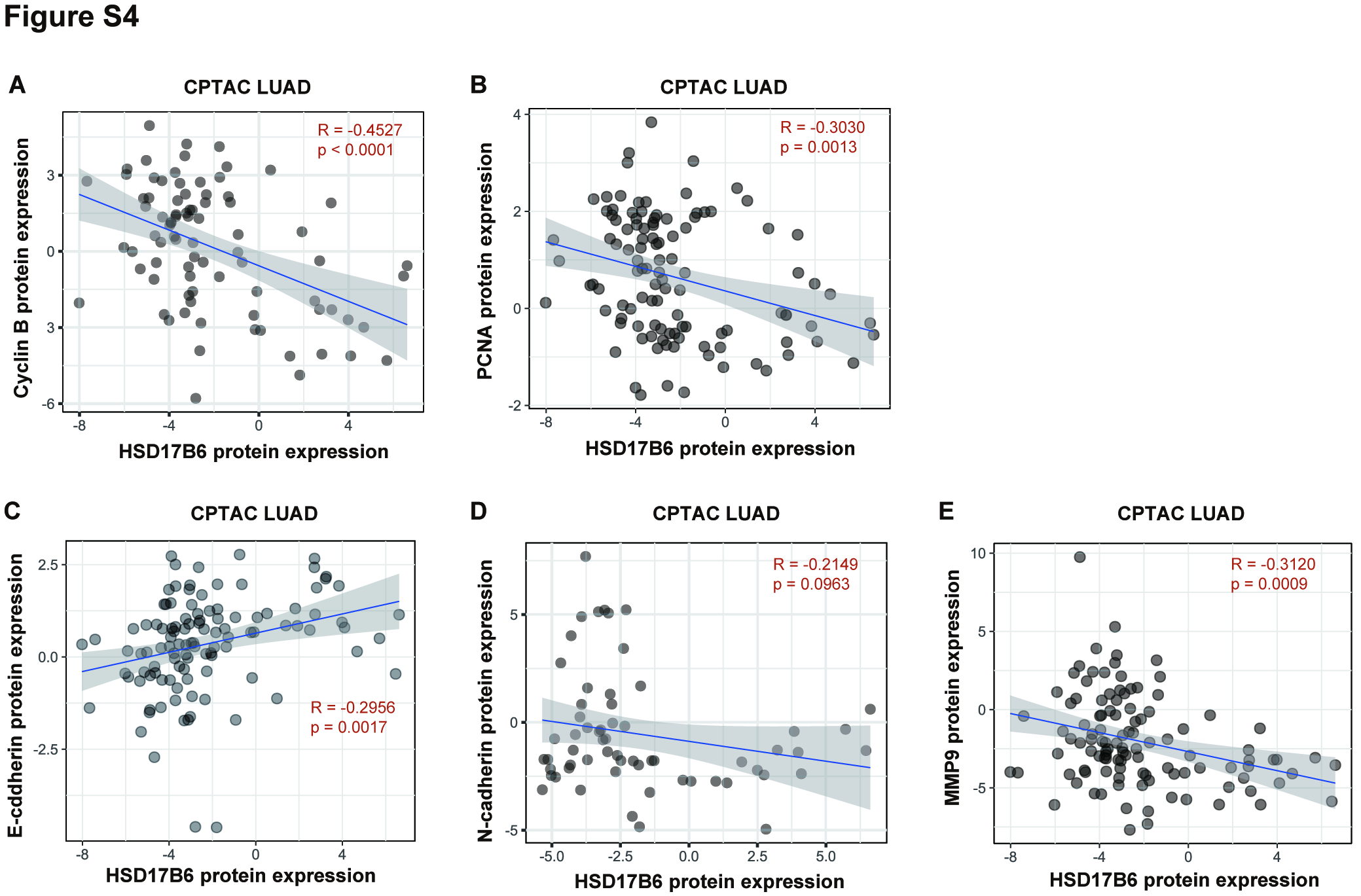

Supplement: Supplementary file 5 — Figure S4 [file 41420_2021_737_MOESM5_ESM.tif]

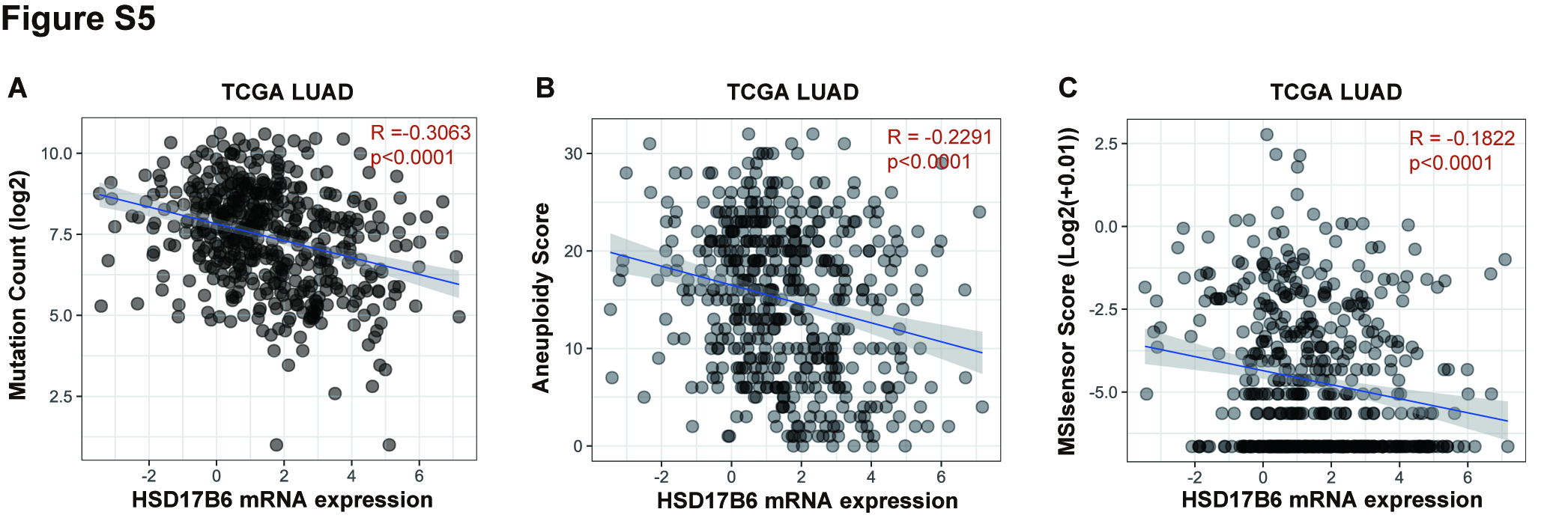

Supplement: Supplementary file 6 — Figure S5 [file 41420_2021_737_MOESM6_ESM.tif]
