# Supplementary material for: HSD17B6 downregulation predicts poor prognosis and drives tumor progression via activating Akt signaling pathway in lung adenocarcinoma
Source: Cell Death Discov. 2021 Nov 8;7:341. doi: 10.1038/s41420-021-00737-0 (PMC8576029; doi:10.1038/s41420-021-00737-0)
Supplement: Supplementary file 7 — Table S1 [file 41420_2021_737_MOESM7_ESM.docx]

**Table S1. Baseline characteristics according to TCGA LUAD Clinical data.**

| **Characteristic** | **levels** | **Low expression of HSD17B6** | **High expression of HSD17B6** | **p** |
| --- | --- | --- | --- | --- |
| **n** |  | 256 | 257 |  |
| **T stage** | T1 | 68 (13.3%) | 100 (19.6%) | 0.027 |
|  | T2 | 150 (29.4%) | 126 (24.7%) |  |
|  | T3 | 26 (5.1%) | 21 (4.1%) |  |
|  | T4 | 11 (2.2%) | 8 (1.6%) |  |
| **N stage** | N0 | 154 (30.7%) | 176 (35.1%) | 0.044 |
|  | N1 | 55 (11%) | 40 (8%) |  |
|  | N2 | 43 (8.6%) | 31 (6.2%) |  |
|  | N3 | 2 (0.4%) | 0 (0%) |  |
| **M stage** | M0 | 175 (47.4%) | 169 (45.8%) | 0.944 |
|  | M1 | 12 (3.3%) | 13 (3.5%) |  |
| **Pathologic stage** | Stage I | 122 (24.2%) | 152 (30.1%) | 0.010 |
|  | Stage II | 66 (13.1%) | 55 (10.9%) |  |
|  | Stage III | 54 (10.7%) | 30 (5.9%) |  |
|  | Stage IV | 12 (2.4%) | 14 (2.8%) |  |
| **Primary therapy xxoooutoutcome outcome** | PD | 44 (10.3%) | 24 (5.6%) | 0.012 |
| **outcome** | SD | 22 (5.2%) | 15 (3.5%) |  |
|  | PR | 2 (0.5%) | 4 (0.9%) |  |
|  | CR | 143 (33.6%) | 172 (40.4%) |  |
| **Gender** | Female | 121 (23.6%) | 155 (30.2%) | 0.004 |
|  | Male | 135 (26.3%) | 102 (19.9%) |  |
| **Race** | Asian | 2 (0.4%) | 5 (1.1%) | 0.309 |
|  | Black or African American | 30 (6.7%) | 22 (4.9%) |  |
|  | White | 194 (43.5%) | 193 (43.3%) |  |
| **Age** | <=65 | 135 (27.3%) | 103 (20.9%) | 0.009 |
|  | >65 | 114 (23.1%) | 142 (28.7%) |  |
| **Smoker** | No | 27 (5.4%) | 47 (9.4%) | 0.018 |
|  | Yes | 222 (44.5%) | 203 (40.7%) |  |

Note: **n (%)**
