# Supplementary material for: HSD17B6 downregulation predicts poor prognosis and drives tumor progression via activating Akt signaling pathway in lung adenocarcinoma
Source: Cell Death Discov. 2021 Nov 8;7:341. doi: 10.1038/s41420-021-00737-0 (PMC8576029; doi:10.1038/s41420-021-00737-0)
Supplement: Supplementary file 8 — Table S2 [file 41420_2021_737_MOESM8_ESM.docx]

**Table S2. Common HALLMARKs associated with HSD17B6 expression in lung adenocarcinoma datasets.**

|  | TCGA LUAD | |  | GSE68465 | |  | GSE72094 | | |
| --- | --- | --- | --- | --- | --- | --- | --- | --- | --- |
|  | ES | FDR q |  | ES | FDR q |  | ES | | FDR q |
| HALLMARK_MYOGENESIS | 0.4503 | 0.0000 | | 0.3029 | 0.0372 | | | 0.3426 | 0.0061 |
| HALLMARK_BILE_ACID_METABOLISM | 0.4417 | 0.0002 | | 0.4691 | 0.0006 | | | 0.4724 | 0.0000 |
| HALLMARK_COAGULATION | 0.4354 | 0.0002 | | 0.4044 | 0.0034 | | | 0.3588 | 0.0055 |
| HALLMARK_UV_RESPONSE_DN | 0.4348 | 0.0003 | | 0.4661 | 0.0012 | | | 0.3680 | 0.0031 |
| HALLMARK_KRAS_SIGNALING_UP | 0.4272 | 0.0003 | | 0.3168 | 0.0331 | | | 0.2688 | 0.0693 |
| HALLMARK_HEME_METABOLISM | 0.3658 | 0.0039 | | 0.3760 | 0.0041 | | | 0.3520 | 0.0038 |
| HALLMARK_P53_PATHWAY | 0.3378 | 0.0089 | | 0.3738 | 0.0051 | | | 0.3011 | 0.0225 |
| HALLMARK_UV_RESPONSE_UP | -0.2709 | 0.0450 | | -0.3179 | 0.0152 | | | -0.3086 | 0.0255 |
| HALLMARK_PI3K_AKT_MTOR_SIGNALING | -0.3015 | 0.0247 | | -0.3605 | 0.0068 | | | -0.3320 | 0.0240 |
| HALLMARK_SPERMATOGENESIS | -0.3438 | 0.0032 | | -0.4177 | 0.0004 | | | -0.3682 | 0.0032 |
| HALLMARK_MITOTIC_SPINDLE | -0.4137 | 0.0000 | | -0.4645 | 0.0000 | | | -0.4554 | 0.0000 |
| HALLMARK_OXIDATIVE_PHOSPHORYLATION | -0.4348 | 0.0000 | | -0.3378 | 0.0048 | | | -0.3565 | 0.0027 |
| HALLMARK_DNA_REPAIR | -0.4746 | 0.0000 | | -0.4428 | 0.0000 | | | -0.4537 | 0.0000 |
| HALLMARK_GLYCOLYSIS | -0.5032 | 0.0000 | | -0.4011 | 0.0004 | | | -0.4538 | 0.0000 |
| HALLMARK_UNFOLDED_PROTEIN_RESPONSE | -0.5586 | 0.0000 | | -0.5234 | 0.0000 | | | -0.5382 | 0.0000 |
| HALLMARK_MTORC1_SIGNALING | -0.5592 | 0.0000 | | -0.5548 | 0.0000 | | | -0.5185 | 0.0000 |
| HALLMARK_G2M_CHECKPOINT | -0.7281 | 0.0000 | | -0.7077 | 0.0000 | | | -0.6865 | 0.0000 |
| HALLMARK_MYC_TARGETS_V1 | -0.7285 | 0.0000 | | -0.6719 | 0.0000 | | | -0.6599 | 0.0000 |
| HALLMARK_E2F_TARGETS | -0.7902 | 0.0000 | | -0.7400 | 0.0000 | | | -0.7247 | 0.0000 |
| HALLMARK_MYC_TARGETS_V2 | -0.7982 | 0.0000 | | -0.7414 | 0.0000 | | | -0.7928 | 0.0000 |

Note:

ES: enrichment score

FDR q: False Discovery Rate q value
